# A case of conventional treatment failure in visceral leishmaniasis: leukocyte distribution and cytokine expression in splenic compartments

**DOI:** 10.1186/1471-2334-14-491

**Published:** 2014-09-09

**Authors:** Washington LC dos-Santos, Carla Pagliari, Lina G Santos, Valter A Almeida, Thiago LV e Silva, João de J Coutinho, Tulio Souza, Maria IS Duarte, Luiz AR de Freitas, Carlos HN Costa

**Affiliations:** Fundação Oswaldo Cruz, Centro de Pesquisas Gonçalo Moniz, Salvador, BA Brazil; Faculdade de Medicina, Universidade de São Paulo, São Paulo, SP Brazil; Universidade Federal do Piauí, Instituto de Doenças Tropicais Natan Portela, Teresina, PI Brazil; Hospital Aliança, Salvador, BA Brazil

**Keywords:** Visceral leishmaniasis, Spleen, Cytokines, Leukocyte populations, *Leishmania infantum*, *Leishmania chagasi*

## Abstract

**Background:**

In this paper we study the distribution of leukocyte populations and of cytokine-producing cells in the spleen of a patient with visceral leishmaniasis resistant to clinical treatment. It is the first attempt to compare the distribution of leukocyte populations and cytokine-producing cells in the splenic compartments of a patient with visceral leishmaniasis with those observed in patients without the disease.

**Case presentation:**

A 25-year-old male, farmer, was hospitalized on several occasions with diagnosis of visceral leishmaniasis and received all recommended treatments for the disease with only transient improvement followed by relapse. He was eventually subjected to splenectomy in order to control the effects of hypersplenism and to potentially overcome infection. After surgery and combined chemotherapy, the disease evolved to cure. In comparison with the spleens of the other two patients without visceral leishmaniasis, an increase was observed in the CD4/CD8 ratio and in the number of IL-10- and FoxP3-producing cells, while the number of IL-17-producing cells was lower in the spleen of the patient with visceral leishmaniasis.

**Conclusion:**

This report confirms previous data on changes in the CD4/CD8 ratio in the spleens of patients with visceral leishmaniasis. Additionally the data presented herein suggests that splenic FoxP3- and IL-17-producing cells are involved in the chronicity of visceral leishmaniasis.

**Electronic supplementary material:**

The online version of this article (doi:10.1186/1471-2334-14-491) contains supplementary material, which is available to authorized users.

## Background

Visceral leishmaniasis is an infectious disease caused by an intracellular parasite of the species *Leishmania infantum* or *Leishmania donovani*[1]. In vertebrate hosts, the parasite lives in the mononuclear phagocytes of a variety of tissues, mainly the liver, spleen, bone marrow and lymph nodes [2]. Visceral leishmaniasis is characterized by hepatomegaly, splenomegaly, fever, emaciation, anemia and an increased susceptibility to bacterial infection and bleeding [3]. Most patients with visceral leishmaniasis respond to treatment with antimonials, or with second-line therapeutics, such as pentamidine or amphotericin [4]. However, some patients present multiple relapses, resulting in the failure of conventional therapeutic intervention [5]. In some of these cases, splenectomy can be performed as a final therapeutic attempt [5]. The literature contains few reports of therapeutic spleen removal in visceral leishmaniasis, and little is known about the mechanism involved in the control of infection obtained via splenectomy [6]. Among the putative contributing factors associated with splenectomy in patients with visceral leishmaniasis are the reduction of parasite burden and the suppression of hypersplenism, which may cause hematological abnormalities [5].

In this study, the distribution of leucocyte populations and of cytokine-producing cells was investigated, using immunohistochemistry, in the spleen of a patient who steadily progressed to cure following splenectomy, performed as a final attempt to treat visceral leishmaniasis. For comparison purposes, splenic samples were selected from the archives of the Service of Anatomopathology of Aliança Hospital, Salvador, Brazil (a non-endemic area of visceral leishmaniasis), obtained from two patients subjected to splenectomy, one during the surgery to remove a non-lymphoid, benign pancreatic neoplasia and another due to trauma in a car accident.

Four-μm thick splenic tissue sections were stained with hematoxylin and eosin. Immunohistochemistry was also performed using the following antibodies: CD3 (policlonal rabbit), CD4 (4B12), CD8 (C8/144B), CD20 (L26), CD79 (JCB117), CD138 (MI15), all from Dako (Glostrup, Denmark) as well as a homemade polyclonal anti-*Leishmania* antibody [7]. Deparffinization and antigen retrieval was performed using a Dako PT Link (PT100/PT101) high pH module at 97°C for 20 minutes. The DAKO EnVision + HRP kit (Dako Co, Denmark) was used as an amplification system. Diaminobenzidine was used as the chromogen. Sections were counterstained with haematoxylin. Mouse IgG1 isotypes unrelated to the antigens were used as a negative control to replace the primary antibody. Cytokine detection was performed using these primary antibodies: MAB 285 (IFNγ), AF-210-NA (TNFα), AF-217-NA (IL10), AF-206-NA (IL6), AF-317-NA (IL17) and AF-204-NA (IL4) (RD Systems), as well as 14-4776-82 (Foxp3 from E-Bioscience), in accordance with the following protocol: Deparaffinization and rehydration were performed in xylene, followed by a decreasing series of ethanol solutions and the blockage of endogenous tissue peroxidase in 3% hydrogen peroxide. Antigen unmasking was performed using a heat-induced antigen retrieval method in a water bath with Retrieval Buffer (Dako Corporation, Carpinteria CA, USA) for 25 minutes at 95°C, pH 9.0. Next, the sections were incubated in a saponin solution (0.1% in PBS 0.01 M, pH7.4) for 10 minutes at room temperature, followed by incubation in skim milk 10% for 30 minutes and a final incubation with the primary antibodies diluted in 1% bovine albumin – PBS solution, overnight at 4°C. Specific antibody binding was detected using a second antibody and the LSAB system (Dako Corporation, Carpinteria CA, USA, K690) for 30 minutes at 37°C. All reactions were developed using a 3′3 diaminobenzidine chromogen solution and counterstained with Harris hematoxilin.

## Case presentation

**Figure 1:**
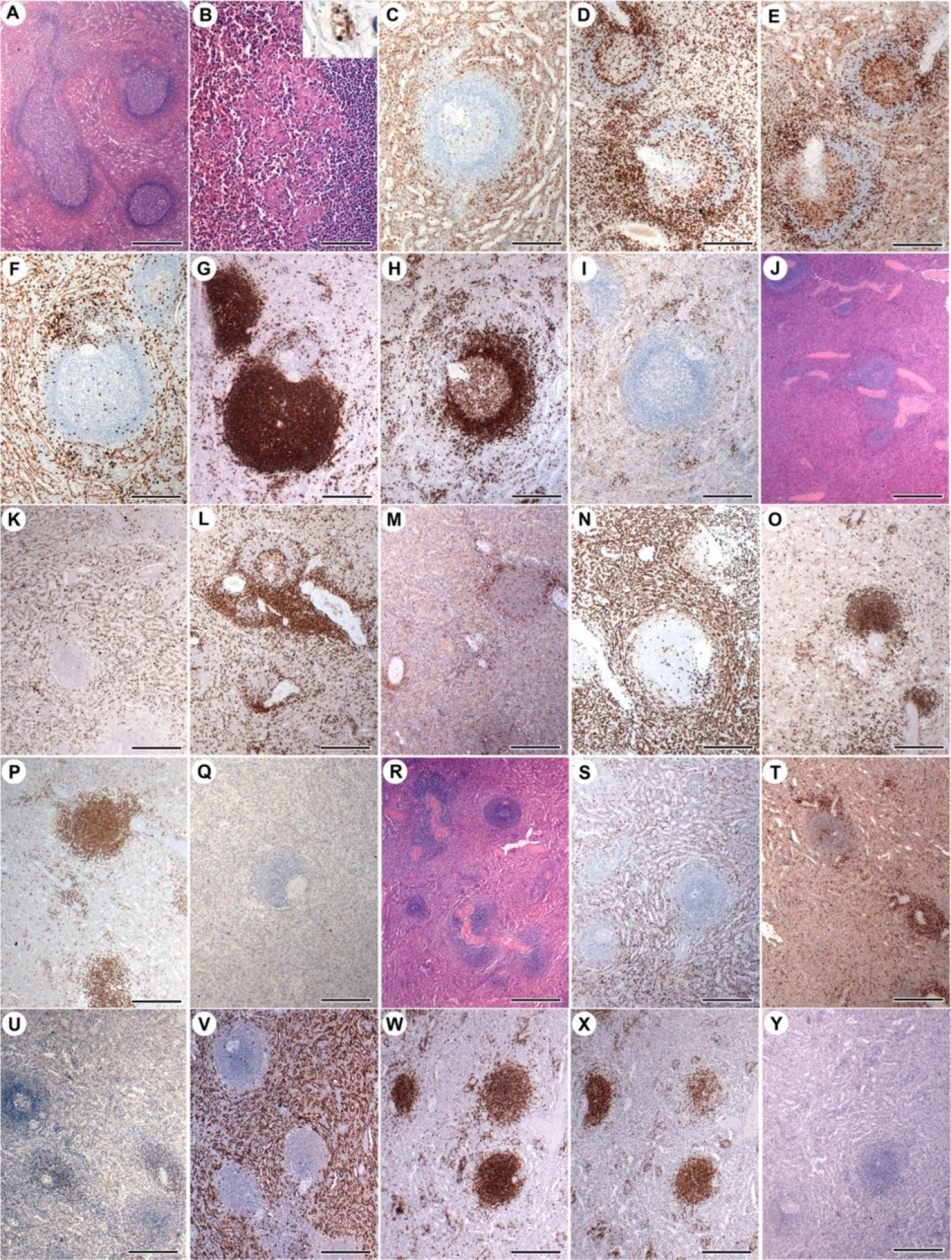
**Parasitism, histological changes and leukocyte populations in the spleen of a patient with chronic relapsing visceral leishmaniasis (A-I) and of two control patients without the disease (J-Q, control #1; R-Y, control #2): A: large secondary follicles; B: ill-formed granuloma; insert: amastigote-containing macrophages.** Leukocyte populations: **C**, **K**, **S**: CD68+ macrophages; **D**, **L**, **T**: CD3+ T lymphocytes; **E**, **M**, **U**: CD4+ T lymphocytes; **F**, **N**, **V**: CD8+ T lymphocytes; **G, O, W**: CD20+ B lymphocytes; **H**, **P**, **X**: CD79α + B lymphocytes; **I**, **Q**, **Y**: CD138+ plasma cells. (figures **A**, **J** and **R**, bar = 600 μm; figures **B**-**I**, **K**-**Q** and **S**-**Y**, bar = 300 μm).

A 25-year-old farmer, male, suffered from recurrent episodes of fever and asthenia, mucocutaneous pallor and abdominal distention. He was hospitalized on several occasions between 2009-2011 with a consistent diagnosis of visceral leishmaniasis, as confirmed by laboratory testing including the finding of amastigotes of the parasite in bone marrow smears. The patient received all recommended treatments for American visceral leishmaniasis, including pentavalent antimony (Gloucantime®), amphotericin B and pentamidine with only transient symptomatic improvement followed by relapse. He was eventually admitted to the Getúlio Vargas Hospital (Teresina, Piauí State, Brazil) in January 2012. Clinical examination revealed mucocutaneous pallor (2+/4+), fever (38.5°C), dehydration (2+/4+), a distended abdomen with mild pain upon deep palpation and splenomegaly (8 cm below the left costal margin). Laboratory tests detected anemia, leucopenia, hypergammaglobulinemia and high levels of ESR and CRP (Table 1). On May 2, 2012, a splenectomy was performed to control the effects of hypersplenism and to potentially overcome infection. After surgery and combined chemotherapy, the disease evolved to cure and the patient remained asymptomatic with improvement observed in laboratory parameters (Table 1). The *Leishmania* isolates were investigated with respect to resistance to the antiparasitic drugs Glucantime, amphotericin and pentamidine, with no resistance pattern observed.The spleen weighed 1,860 g, and measured 25.0 × 17.0 × 9.0 cm. The capsule was thin and transparent. The cut surface was deep red with areas of necrosis and hemorrhage. Histologically, the white pulp was well-organized with large-sized secondary lymphoid follicles surrounded by well-defined marginal zones (Figure 1A). The periarteriolar lymphoid sheaths (PALS) were clearly evident. The red pulp was hyperplastic with prominent cords, dilated sinusoids and an increased amount of fusiform cells. Amastigotes containing macrophages, small aggregates of plasma cells, occasional erythrocytes containing macrophages, as well as ill-defined granulomas with infected macrophages, were observed among the splenic cords (Figure 1B and insert).

**Table 1:** **Laboratory data of the patient with visceral leishmaniasis before (7/28/2009 to 4/10/2012) and after (5/15/2012) splenectomy**

LABORATORY	DAY						
TEST	7/28/2009	8/19/2010	1/13/2011	5/13/2011	1/12/2012	4/10/2012	5/15/2012
Hematocrit	31	36	36	24	21	27	38
Hemoglobin	9.1	11.7	11.9	8.2	6.6	8.2	11
WBC count	2100	2360	2500	2380	1410	2280	5500
Lymphocyte count	588	1133	1200	1261	705		
Platlet count	136000	185000	210000	150000	154000	70300	570000
Urea	31	23	22	31	32	38	
Creatinine	1.1	0.9	0.8	1.2	1,3	1,4	
ALT	20	41	59		54		
AST	32	10	25		17		
ESR (1 h/2 h)	80/110					109/124	50/70
CRP					R	NR	NR
Bilirrubin (direct)	0.7 (0.3)	1.0 (0.5)				0.8 (0.4)	
Total Protein	9.2	5.2	8.6			7.1	
Albumin	2.9	3.5	2.8			3.2	
PT/P activity	41 s/22%		18 s/54%	28 s/29%		14.6 s/75%	
RNI	1.9		1.47	2.12		1.15	
Glucose			69		79	93	
ALP	80	176				213	
GGT						68	
Rapid test for HIV	NR	NR			NR		
Leishmania search in bone marrow smears	POS	POS	POS	POS	POS	NEG	NEG
Rapid test for VL	POS			POS	NEG	NEG	NEG
IFR for *Leishmania*	1/160	NEG					
HAV						NEG	
HCV					POS	NEG	
HBsAg					NEG	NEG

Along with the spleen, a perisplenic lymph node collected during the surgery was received. The lymph node measured 1.2 × 0.7 cm. The lymph node capsule was infiltrated by lymphocytes and *Leishmania*-infected macrophages. The lymph node structure was normal, the marginal sinus contained few lymphocytes and the lymphoid follicles were mostly small with germinal centers (not shown). The paracortical areas were expanded and partially replaced by large amastigote-containing macrophages, which were also seen in the interior of the follicles and germinal centers. The medullary cords and medullary sinus were barely evident, containing many plasma cells and infected macrophages (not shown).Control #1: A 42-year-old female had her spleen removed in the course of a surgery to resect a solid pseudopapillary (benign) pancreatic tumor. The spleen weighed 83.0 g and measured 10.5 × 6.0 × 3.0 cm. Spleen sections showed small lymphoid follicles with small germinal centers and the mantle zones were not uniformly apparent. Some follicles showed hyalinosis. The red pulp had a predominance of macrophages and lymphocytes with some neutrophils present. Some arterioles presented hyaline arteriolosclerosis (Figure 1J).Control #2: A 31-year-old male underwent splenectomy 3 h after suffering traumatic spleen rupture due to a car accident. The spleen weighed 133 g and measured 10.0 × 7.0 × 3.8 cm. Spleen sections showed a large area of recent hemorrhage, surrounded by normal spleen parenchyma. The lymphoid follicles were small, had small germinal centers, and the mantle zones were not consistently evident. The red pulp sinus were dilated and the red pulp contained many reticular cells, macrophages, lymphocytes and neutrophil aggregates. Some arterioles presented hyaline arteriolosclerosis (Figure 1R).

**Figure 2:**
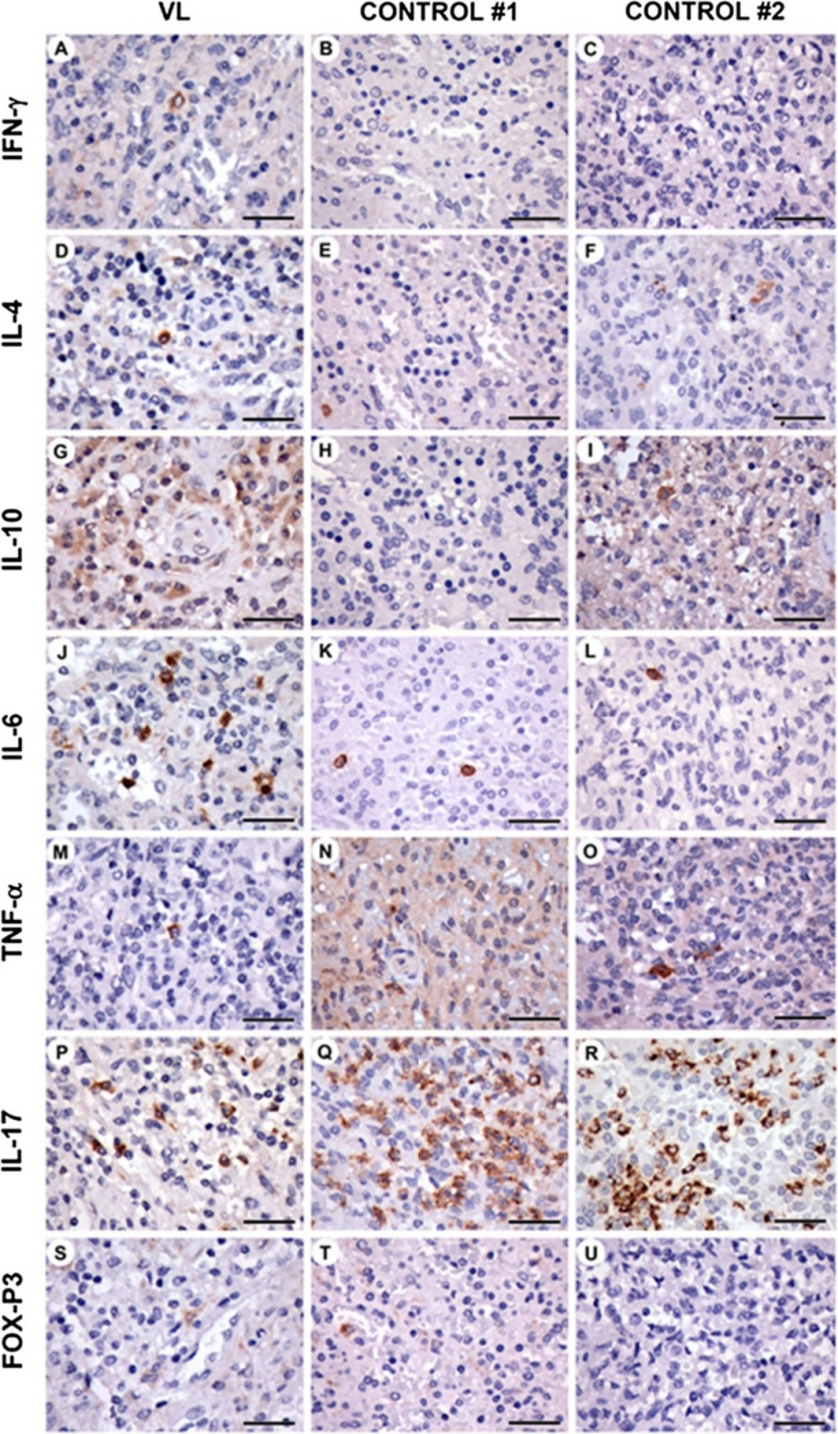
**Cytokine-producing cells in the spleen of a patient with chronic relapsing visceral leishmaniasis, as well as two control patients: A-C: IFNγ; D-F: IL4; G-I: IL-10; J-L: IL-6; M-O: TNFα; P-R: IL17; S-U: Fox-P3.** (bar = 30 μm).

The distribution of the leukocyte populations identified by immunohistochemistry shown in Figure 1 was similar to what is described in the literature [8] in all three cases studied. Immunohistochemistry revealed very few isolated mononuclear cells producing IFN-γ (Figure 2A-C), IL-4 (Figure 2D-F), IL-10 (Figure 2G-I) and TNF-α (Figure 2M-O), mostly in the red pulp. A large number of IL-6 (Figure 2J-L) and IL-17 (Figure 2P-R)-producing cells were observed in the red pulp and in the PALS. Fox-P3 (Figure 2S-U)-expressing cells were found in the red pulp, PALS and in marginal zones.Compared to the spleens of the other two patients, the spleen of the patient with visceral leishmaniasis had increased numbers of: macrophages in the lymphoid follicles, CD4 T cells in the red pulp (Figure 1E compare with 1M and 1U), B cells in the follicles and in the marginal zones (Figure 1G and H, compare with 1O-P and 1W-X), and plasma cells in the red pulp and in the marginal zones (Figure 1I, compare with 1Q and 1Y). In addition, the density of IL10-producing cells was higher in the spleen of the patient with visceral leishmaniasis (Figure 2G-I). This patient had generally fewer IL17-producing cells (Figure 2P-R). The density of Fox-P3-producing cells was higher in the red pulp of the patient with visceral leishmaniasis in comparison to what was observed in the other patients (Figure 2S-U).

To the best of our knowledge, the literature contains only one other report by Nylén and collaborators [9] on the distribution of leukocyte populations in the spleen, as well as cytokine production by human splenocytes in visceral leishmaniasis. In this study, the authors, using flow cytometry and RT-PCR on splenic cell aspirate, were not able to demonstrate an increased number of Fox-P3 positive cells in the spleens of patients with acute visceral leishmaniasis (with 2-3 weeks of duration). By contrast, the patient reported herein had a chronic form of visceral leishmaniasis that lasted for more than three years. An increased density of IL10 and Fox-P3-producing cells was observed in the red pulp, as well as in the follicles and in the marginal zones in comparison with the spleens of the patients without visceral leishmaniasis. Hence, these findings suggest the possibility that Fox-P3 cells also accumulate in chronic visceral leishmaniasis, similar to what occurs in cutaneous leishmaniasis and other chronic diseases. Conversely, the density of IL-17-expressing cells was generally lower in the spleen of patient with visceral leishmaniasis patient in comparison to the spleens of the other two patients. The high density of both IL-17- and IL-6-producing cells observed in the spleens of all three patients was notable. Such high IL-17 and IL-6 production in the spleen supports the notion that the spleen plays an important role in the surveillance of blood-borne infections. In fact, IL-17, together with IL-6, participate in an axis of inflammation-promoting cytokines involved in the innate response to bacteria and *Leishmania*[10, 11]. Pitta and colleagues [12] showed that peripheral blood mononuclear cells from patients with visceral leishmaniasis produced lower levels of IL-17 after stimulation with *Leishmania* antigens than the cells of individuals resistant to the disease. These authors suggest that susceptibility to visceral leishmaniasis may be associated with an impairment in Th-17 cell differentiation upon stimulation with IL-1β and IL-6. Nylén and colleagues [9] also observed an increase in the CD4/CD8 ratio, which decreased in response to amphotericin treatment. In the patient with visceral leishmaniasis presented herein, the CD4/CD8 ratio was found to be higher in the different spleen compartments in comparison to the other patients.

In addition to the findings described above, the present case confirms extensive reports on plasmacytosis in association with visceral leishmaniasis. Furthermore, we have demonstrated that plasma cell accumulation is more pronounced in the red pulp and in the marginal zones, which suggests that some of these cells may not reach normal maturation in the PALS.

## Conclusion

This report confirms previous data on changes in the CD4/CD8 ratio in the spleens of patients with visceral leishmaniasis. It also suggests that splenic FoxP3- and IL-17-producing cells are involved in the chronicity of visceral leishmaniasis. The authors feel that the data presented herein may contribute to the understanding of disease progression and host parasite interaction in severe forms of visceral leishmaniasis.

### Consent

Written informed consent was obtained from the patient for publication of this Case report and any accompanying images. A copy of the written consent is available for review by the Editor of this journal.

## Authors’ original submitted files for images

Below are the links to the authors’ original submitted files for images. Authors’ original file for figure 1 Authors’ original file for figure 2
