# Supplementary figures and images for: A case of conventional treatment failure in visceral leishmaniasis: leukocyte distribution and cytokine expression in splenic compartments
Source: BMC Infect Dis. 2014 Sep 9;14:491. doi: 10.1186/1471-2334-14-491 (PMC4175220; doi:10.1186/1471-2334-14-491)

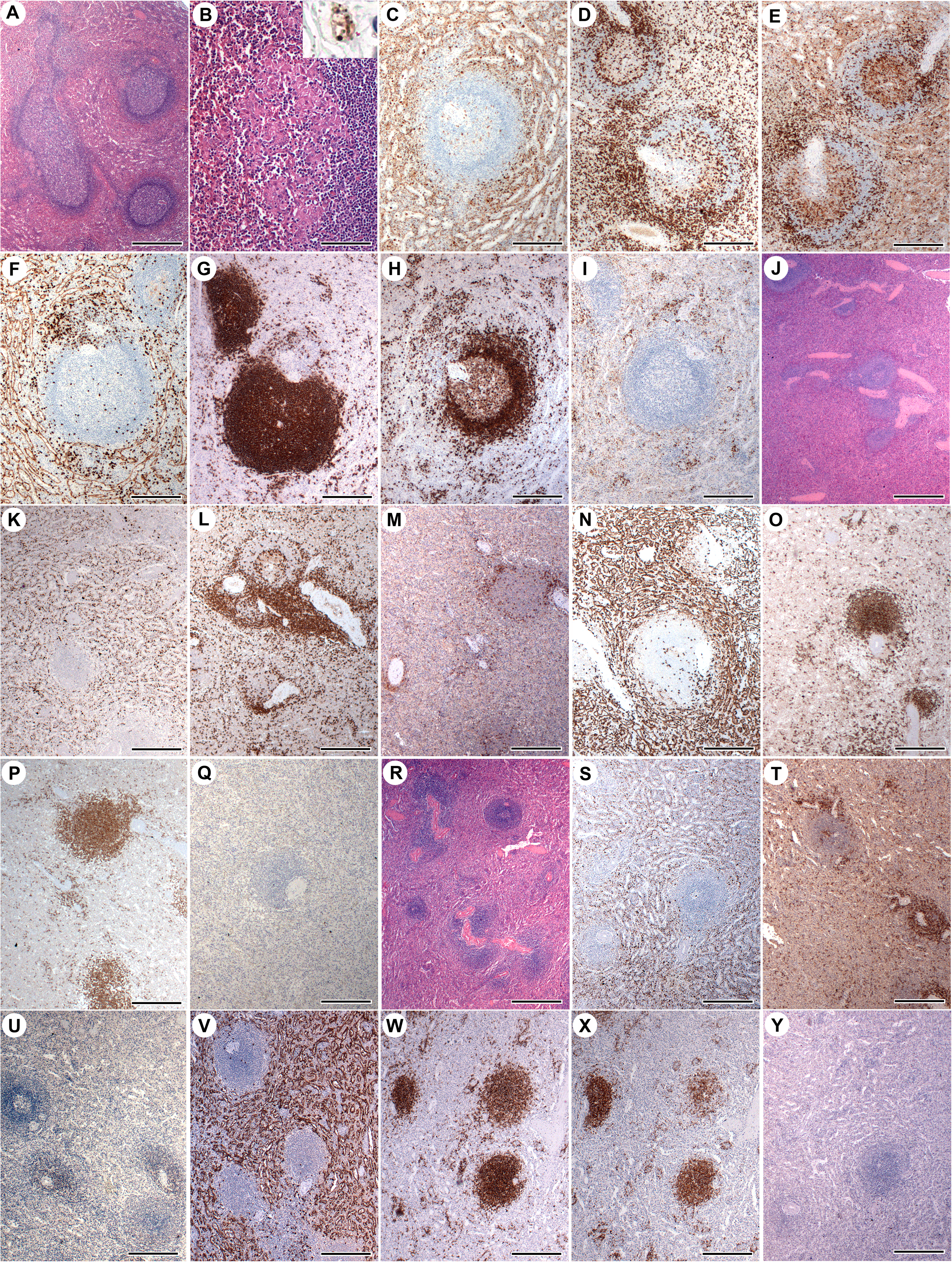

Supplement: Supplementary file 1 — Authors’ original file for figure 1 [file 12879_2014_3824_MOESM1_ESM.tiff]

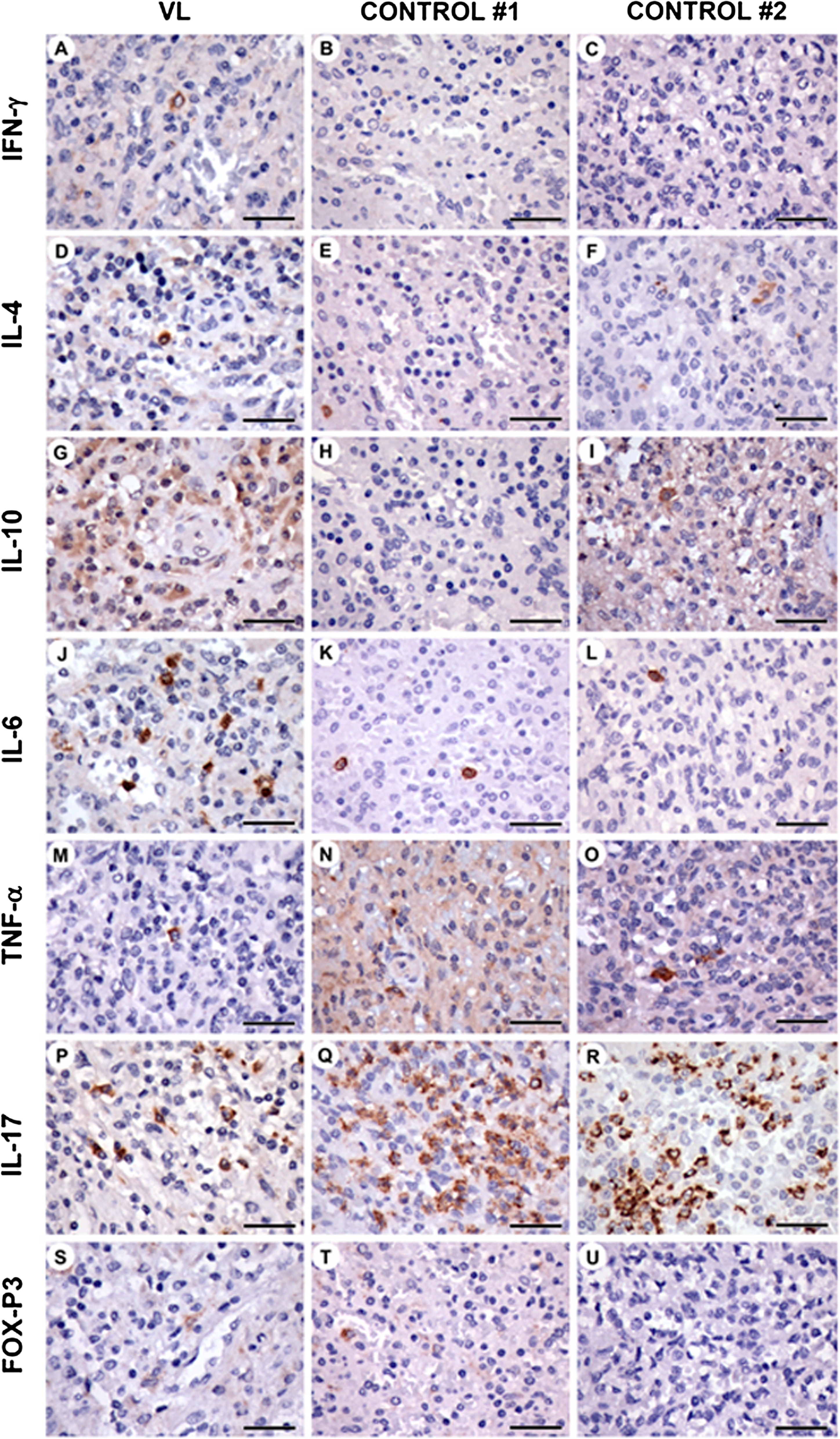

Supplement: Supplementary file 2 — Authors’ original file for figure 2 [file 12879_2014_3824_MOESM2_ESM.tif]
